# Supplementary material for: Differentiated historical demography and ecological niche forming present distribution and genetic structure in coexisting two salamanders (Amphibia, Urodela, Hynobiidae) in a small island, Japan
Source: PeerJ. 2022 Apr 28;10:e13202. doi: 10.7717/peerj.13202 (PMC9057287; doi:10.7717/peerj.13202)
Supplement: Supplemental Information 2 — Asterisks indicate that Group A and Group B were discovered syntopically. [file peerj-10-13202-s002.docx]

| **Group** | **Locality** | **N** | **Nh** | **Haplotype No.** | **DDBJ accession No.** | **Source** |
| --- | --- | --- | --- | --- | --- | --- |
| Group A | Tsutsu | 3 | 2 | A2, A3(2) | LC638502-LC638504 | This study |
|  | Tsutsuse | 2 | 1 | A2(2) | LC156103, LC156104 | Niwa et al. (2021) |
|  | Azamo | 3 | 1 | A3(3) | LC156105-LC156107 | Niwa et al. (2021) |
|  | Agami | 2 | 2 | A3, A4 | LC638505, LC638506 | This study |
|  | Kuwa | 1 | 1 | A3 | LC638507 | This study |
|  | Taterasan1 | 1 | 1 | A3 | LC638508 | This study |
|  | Taterasan2 | 1 | 1 | A3 | LC638509 | This study |
|  | Uchiyama | 2 | 2 | A5, A6 | LC638510, LC638511 | This study |
|  | Kunehama | 2 | 1 | A3(2) | LC638512, LC638513 | This study |
|  | Shiine | 1 | 1 | A7 | LC638514 | This study |
|  | Kitazato1 | 2 | 2 | A1, A3 | LC156108, LC156109 | Niwa et al. (2021) |
|  | Kitazato2 | 2 | 2 | A3, A8 | LC638515, LC638516 | This study |
|  | Kitazato3 | 1 | 1 | A9 | LC638517 | This study |
|  | Kitazato4 | 1 | 1 | A10 | LC638518 | This study |
|  | Shimobaru1 | 2 | 2 | A3, A11 | LC156110, LC156111 | Niwa et al. (2021) |
|  | Shimobaru2 | 2 | 2 | A12, A13 | LC583751, LC583752 | Niwa et al. (2021) |
|  | Shimobaru3 | 2 | 2 | A14, A15 | LC583753, LC583754 | Niwa et al. (2021) |
|  | Shimobaru4 | 2 | 1 | A3(2) | LC638519, LC638520 | This study |
|  | Shimobaru5 | 1 | 1 | A2 | LC638521 | This study |
|  | Sumo1* | 4 | 3 | A3(2), A10, A22 | LC156112, LC156113, LC156115, LC156116 | Niwa et al. (2021) |
|  | Sumo2 | 2 | 2 | A3, A17 | LC638522, LC638523 | This study |
|  | Kashi | 3 | 2 | A3(2), A16 | LC638524-LC638526 | This study |
|  | Mikata* | 2 | 2 | A10, A16 | LC638527, LC638528 | This study |
|  | Kechi | 3 | 3 | A3, A16, A20 | LC638529-LC638531 | This study |
|  | Mine* | 3 | 3 | A3, A17, A22 | LC638532-LC638534 | This study |
|  | Shishimi* | 1 | 1 | A24 | LC156132 | Niwa et al. (2021) |
|  | Kaidokoro* | 3 | 3 | A18, A19, A27 | LC156123-LC156125 | Niwa et al. (2021) |
|  | Seta | 1 | 1 | A19 | LC638535 | This study |
|  | Mitake2* | 2 | 2 | A23, A24 | LC638536, LC638537 | This study |
|  | Saozaki | 2 | 1 | A26(2) | LC638538, LC638539 | This study |
|  | Sago1 | 2 | 2 | A21, A24 | LC638540, LC638541 | This study |
|  | Sago2 | 1 | 1 | A24 | LC638542 | This study |
|  | Shitaru | 1 | 1 | A3 | LC638543 | This study |
|  | Sasuna* | 1 | 1 | A19 | LC638544 | This study |
|  | Oshika* | 2 | 2 | A17, A24 | LC638545, LC638546 | This study |
|  | Kin* | 4 | 4 | A17, A24, A28, A29 | LC156133-LC156135, LC156137 | Niwa et al. (2021) |
|  | Shushi | 3 | 3 | A3, A19, A25 | LC638547-LC638549 | This study |

| **Group** | **Locality** | **N** | **Nh** | **Haplotype No.** | **DDBJ accession No.** | **Source** |
| --- | --- | --- | --- | --- | --- | --- |
| Group B | Sumo1* | 3 | 1 | B1(3) | LC156114, LC638550, LC638551 | Niwa et al. (2021), this study |
|  | Mikata* | 2 | 2 | B1, B2 | LC638552, LC638553 | This study |
|  | Itose | 3 | 2 | B3(2), B4 | LC583773, LC156117 | Niwa et al. (2021) |
|  | Nii | 1 | 1 | B3 | LC638554 | This study |
|  | Otsuna | 2 | 1 | B3(2) | LC638555, LC638556 | This study |
|  | Yoshida1 | 2 | 2 | B3, B5 | LC156118, LC156119 | Niwa et al. (2021) |
|  | Yoshida2 | 1 | 1 | B7 | LC638557 | This study |
|  | Mine* | 2 | 2 | B3, B4 | LC496473, LC430988 | Niwa et al. (2021) |
|  | Kushi | 1 | 1 | B4 | LC638558 | This study |
|  | Shishimi* | 1 | 1 | B1 | LC638559 | This study |
|  | Kaidokoro* | 3 | 1 | B6(3) | LC583775 | Niwa et al. (2021) |
|  | Mitake1 | 1 | 1 | B8 | LC638560 | This study |
|  | Mitake2* | 3 | 1 | B6(3) | LC638561-LC638563 | This study |
|  | Sasuna* | 2 | 1 | B7(2) | LC638564, LC638565 | This study |
|  | Oshika* | 2 | 2 | B6, B9 | LC638566, LC638567 | This study |
|  | Kin* | 3 | 1 | B7(3) | LC583776 | Niwa et al. (2021) |
|  | Hamakusu | 2 | 1 | B7(2) | LC638568, LC638569 | This study |
